# Total Pain Management and a Malignant Wound: The Importance of Early Palliative Care Referral

**DOI:** 10.7759/cureus.20678

**Published:** 2021-12-25

**Authors:** Catarina Faria, Vanessa Branco, Pedro Ferreira, Cristina Gouveia, Sara Trevas

**Affiliations:** 1 Department of Internal Medicine, Hospital São Francisco Xavier, Lisbon, PRT

**Keywords:** palliative care, existential suffering, total pain, malignant wound, male breast cancer

## Abstract

Breast cancer may metastasize to the lung, liver, bone, brain, and skin, with especially high rates of metastasis to skin sites.These skin metastases are called malignant wounds. Patients with malignant wounds often report multiple symptoms, and pain is one of the most common and distressing among them. Despite the availability of multiple guidelines about treatment to relieve pain, almost half of all cancer patients still receive inappropriate care for pain. A multidisciplinary approach can improve outcomes in terms of symptom control and quality of life and enable the detection of previously unmet needs of both patients and caregivers. Palliative care is a multidisciplinary therapy that aims to alleviate physical, psychological, and emotional suffering in patients at any stage of the disease.

We present the case of a 53-year-old male with a three-year history of stage IV breast cancer. He was admitted to the internal medicine ward in July 2021 with uncontrolled pain related to a malignant wound in the left hemithorax. This was a case with physical, emotional, social, and existential factors contributing to severe pain, necessitating a multidisciplinary approach for adequate relief. Opioid titration and insomnia and anxiety treatment were initiated. Dressing care was applied with metronidazole impregnation and aminocaproic acid for hemorrhagic spots, followed by fat gauze. He was proposed to undergo antalgic radiotherapy, which was unfortunately associated with new onset of symptoms. Psychological support was provided for the patient and his family. We managed to control the pain and stabilize the wound; however, cachexia become evident with the disease progression. In the last week of his life, the patient still believed he would be able to undergo chemotherapy. He died in the emergency room, where he had gone to seek relief for uncontrolled symptoms.

Even though the patient had an incurable disease associated with immense suffering since early 2019, he was only referred to the palliative care team during the last three months of his life. Existential suffering was an important dimension of this patient’s pain and was present until his death despite receiving psychological support. Late referral to palliative care is unfortunately frequent and often associated with poor quality of life and inability to plan or make end-of-life care decisions. Radiotherapy was proposed for pain control but was associated with serious side effects. In a palliative care setting, decision-making always needs careful consideration related to benefit versus harm and must involve the patient and his family.

Living with stage IV cancer is an everyday challenge for patients, and clinicians may also find managing such patients very arduous and stressful. Symptoms must be actively studied and evaluated from a multidimensional perspective. Managing expectations throughout this process while maintaining hope is a delicate balancing act and should be undertaken by specialized palliative care teams.

## Introduction

Breast cancer is associated with metastasis to the lung, liver, bone, brain, and skin, with especially high rates of metastasis to skin sites [1]. These skin metastases are known as malignant wounds. It has been reported that malignant wounds occur in 14.5% of patients with advanced cancer [2]. Patients with malignant wounds often report multiple symptoms: pain, distress from odor and exudate, decreased self-esteem, distress, loss of dignity, and associated decreased quality of life [2]. In fact, pain is one of the most common and distressing symptoms reported by cancer patients, especially those with metastatic disease. Over 80% of metastatic cancer patients suffer from pain caused mostly by direct tumor infiltration [3]. Many guidelines have been published since 1986 in an effort to relieve cancer-related pain and thereby decrease the prevalence of inadequately treated pain; however, almost half of all cancer patients still receive inadequate care for pain [4].

It has been proven that the early integration of palliative actions can address the complaints reported by patients, including those related to symptoms, quality of life, depression, coping, and understanding of the disease, in addition to having a favorable impact on overall survival rates [5]. A multidisciplinary approach can improve outcomes in terms of symptom control and quality of life and enable the detection of previously unmet needs of both patients and caregivers [6]. Palliative care is a multidisciplinary therapy that aims to alleviate physical, psychological, and emotional suffering in patients at any stage of the disease [5].

## Case presentation

We present the case of a 53-year-old male with stage IV breast cancer. He was a former intravenous drugs user and an active smoker with a past medical history of HIV 1 chronic infection suppressed by antiretroviral therapy, hepatitis C virus chronic infection in remission after treatment, asymptomatic emphysema, and chronic periphery arterial disease.

**Figure 1:**
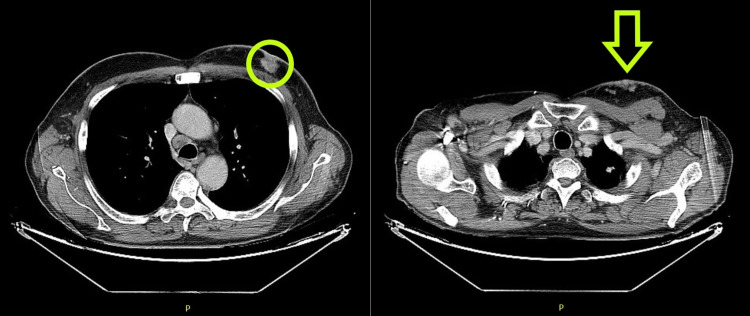
Chest CT on the left showing primitive breast cancer (green circle) and on the right demonstrating skin metastasis (green arrow) CT: computed tomography

**Figure 2:**
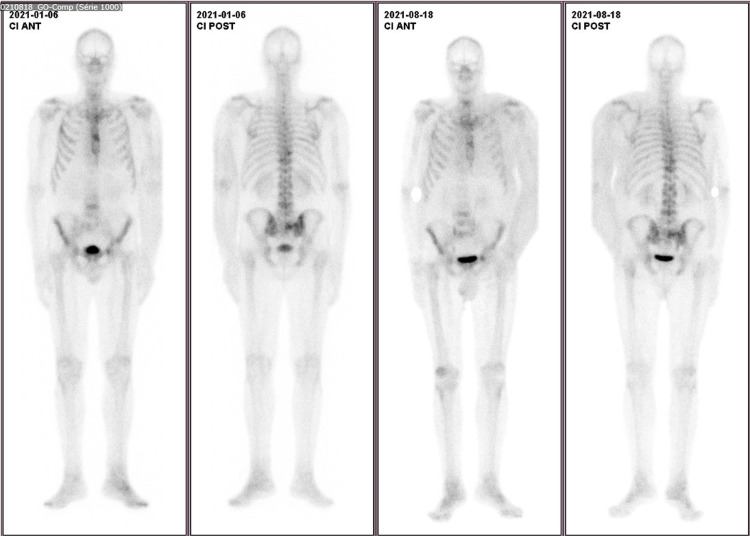
Bone scintigraphy with metastasis in the spine, iliac crest, pelvis, sternum, and ribs

The patient was diagnosed with stage IV breast cancer in April 2019 with lymphatic skin and bone metastasis. His Eastern Cooperative Oncology Group (ECOG) performance status was zero at diagnosis. The tumor was positive for estrogen, progesterone, and HER-2 receptors, and hence was treated with letrozole, trastuzumab, and zoledronic acid until January 2020. By January 2020, the bone disease had progressed, and letrozol was suspended and tamoxifen and chemotherapy with doxorubicin plus cyclophosphamide were initiated, followed by weekly paclitaxel with a good response. Due to new disease progression in January 2021, he was started on fulvestrant and trastuzumab emtansine (TDM1). However, the disease continued to progress, presenting with painful lymphedema of the left arm related to lymph node invasion (deep vein thrombosis was excluded). He also developed new-onset skin lesions, first on the left hemithorax and then spreading to the right, developing a malignant wound associated with severe pain. He was admitted to the internal medicine ward in July 2021 with uncontrolled pain. Figure 1 shows chest CT with primitive lesion and skin metastasis, and Figure 2 shows bone scintigraphy with bone metastasis.

The pain led to constant pressure on the left hemithorax extending to the left shoulder and arm and was classified as 7/10 in intensity. The patient had a malignant wound on the left hemithorax with nodules on the right. The left arm was swollen with exuberant lymphedema. Axillary and supraclavicular nodes were palpable. He was anxious and concerned about the progression of the wound and uncontrolled pain. He also complained about insomnia. He had no other physical symptoms. Even though he knew he had an incurable disease, he still maintained high hopes with regard to possible treatment options, especially since he had always managed to get better with treatment so far. He lived alone and the arm lymphedema interfered with movements and daily activities. He also suffered from a disruption of self-image, and occasionally stated that he could not recognize himself in the mirror. He had three sisters but only one of them lived in Portugal and she just had a newborn baby. He valued his independence and could not bear becoming dependent on others, and mentioned that he developed suicidal thoughts related to fears about relying on others. His mom had died of cancer and this had left a deep trauma in him and his family.

**Figure 3:**
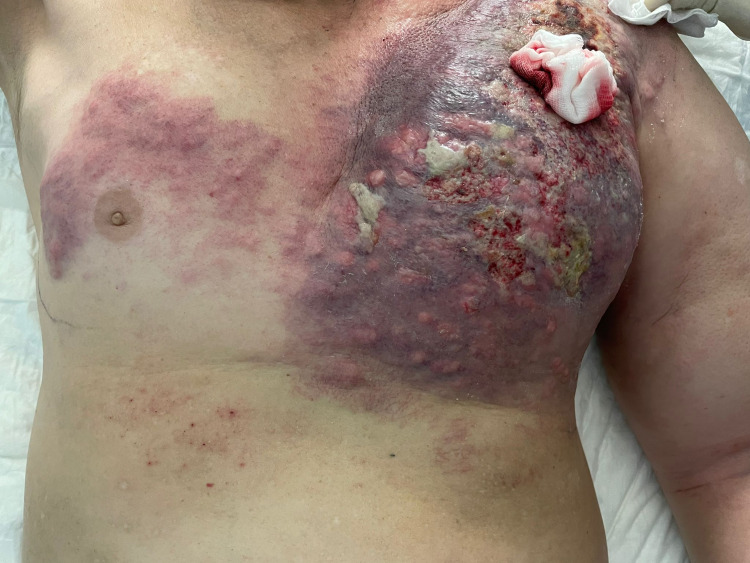
Malignant wound

An opioid titration was made with intravenous morphine with a switch to transdermal fentanyl after stabilization (maximum dose of 75 ug/hour every three days). We also initiated trazodone 50 mg at bedtime and alprazolam 0.25 mg as needed for anxiety. He was advised to undergo 10 sessions of antalgic radiotherapy. Dressing care was applied with metronidazole impregnation and aminocaproic acid for hemorrhagic spots, followed by the application of fat gauze (Figure 3).

After the first 10 sessions of radiotherapy, he presented with xerostomia and odynophagia, and symptomatic treatment was initiated with saliva replacement and sucralfate every six hours, nonsteroidal anti-inflammatory drugs, and mouthwash with lidocaine before every meal.

**Figure 4:**
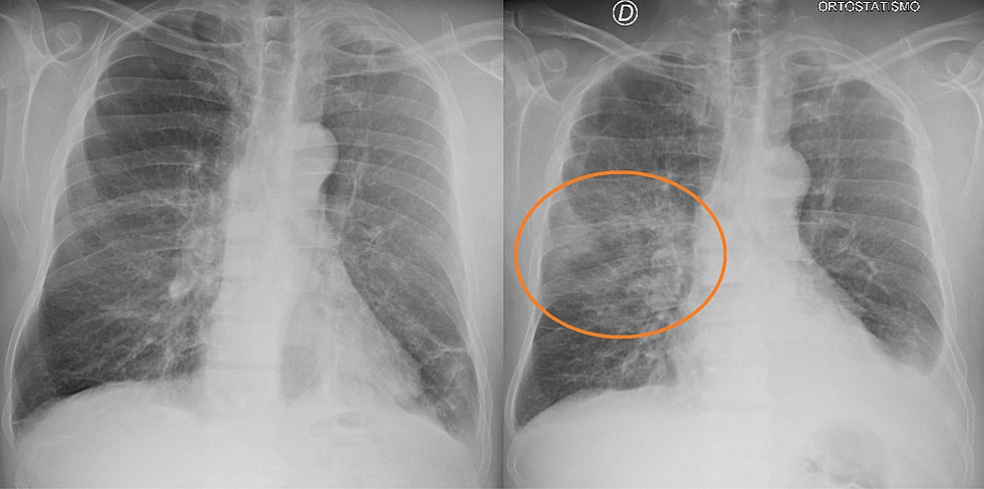
X-ray of the patient before (left) and after (right) radiotherapy X-ray image on the right shows radiation pneumonitis (orange circle)

He also developed cough and dyspnea, and a diagnosis of radiation pneumonitis was made. He was treated with corticosteroids and oxygen supplementation (Figure 4).

Palliative care team support had been requested at admission and psychological support was provided to the patient and his family. The patient showed the team a social media account where he shared the challenges and developments related to his disease with some of his friends. He had created this account after his diagnosis and thought of it as a legacy-building tool. Even though he knew that he had an incurable disease and the aim of the treatment was symptom control, he always believed that the treatment would prolong his life and give him more time.

We managed to control the pain and to stabilize the wound; however, cachexia become evident with progressive disease and he did not meet the clinical criteria for maintaining chemotherapy, as initially proposed by Oncology.

The patient was discharged in September 2021 with arrangements for palliative hospital team support (outpatient evaluation and phone support) as he refused palliative care team support at home. In the last week of life, the patient still believed that he would be able to undergo chemotherapy again. He died at the end of October in the emergency room, where he had gone to seek relief for uncontrolled symptoms.

## Discussion

Symptomatic treatment in palliative care patients goes beyond the physical dimension of the disease. It also should address the emotional and psychological needs of the patient.

Our patient represents a case of total pain with its physical dimension associated with a malignant wound. The emotional dimension was expressed through his anxiety and insomnia. Existential suffering was manifested with ambivalent thoughts, with the knowledge about the unavailability of a permanent cure combined with the hope of getting better. Another important part of the existential dimension was the fear of dependency and this was associated with the personal experience of his mom’s death and worries about his sister’s suffering. Social support was lacking because he lived alone; two of his three sisters were abroad, and the one residing in Portugal had a newborn baby to care for. The disruption of self-image was also noted, and it represents an important challenge in patients with malignant wounds. All of this added layers of complexity to optimal pain management and relief from suffering.

Even though the patient had an incurable disease associated with immense suffering since early 2019, he was only referred to the palliative care team during the last three months of life. This kind of late referral is unfortunately frequent and often associated with poor quality of life and the lack of ability to plan or make end-of-life care decisions [7]. Obstacles to early referral for palliative care are varied, including being unsure of the disease process, the possibility of periods of remission, inadequate communication, and lack of knowledge about palliative care, support, as well as lack of time and team accessibility [3].

Existential suffering was an important aspect of this patient’s pain and psychological support was provided. The rationale behind the approach toward existential distress is that its management can reduce the risk of suicidal ideation, mental disorders, nonadherence to treatment, and low quality of dying and death [8].

The authors would also like to discuss the role of radiotherapy as an antalgic therapy. Cancer pain management must be integrated and involve a multidisciplinary approach. Radiotherapy is often used for optimal pain management, especially in bone metastasis [4]. One important aspect about radiation treatments is that studies have shown that around 20-25% of patients die within two weeks of completing radiation, and nearly 20% of patients who received radiation in the last 30 days of their life spent more than 10 of those days receiving radiation treatment [9]. Moreover, side effects may also occur and contribute to symptom burden as in this case (odynophagia, xerostomia, cough, and dyspnea). In the palliative care setting, decision-making always needs careful considerations about benefit versus harm and must involve the patient and his family.

Another important feature of this case was that the patient had to come to the emergency room during the last hours of his life, despite palliative care team support. Unfortunately, this is also not an uncommon outcome in palliative care patients, and death in Portugal occurs mainly in hospitals. In fact, a study showed that 61.7% of deaths occurred at hospitals/clinics, despite home being referred to as the preferred place of death among the inquired [10]. Several factors may contribute to this outcome. In this case, social frailty was an important factor and, as mentioned above, late referral to palliative care may also have contributed to this, mainly due to the patient's persistent existential suffering.

## Conclusions

We discussed the case of a patient with stage IV male breast cancer complicated by a malignant wound associated with total pain. The difficulties he faced in relieving social and existential suffering may have contributed to his uncontrolled symptoms and, ultimately, his death in the emergency room.

Living with stage IV cancer is an everyday challenge for patients, and clinicians also find managing such patients very difficult. New treatments have improved chances of survival and made it a chronic progressive disease. In caring for such patients, clinicians should engage in a benefit-versus-harm analysis on a case-by-case basis with the aid of multidisciplinary teams; the decision-making should also involve the patient and his family.

Late referral to palliative care teams remains an important obstacle to adequate relief of pain and suffering. Symptoms must be actively studied and evaluated on every dimension (physical, emotional, social, and existential). Pain relief is a human right and social and existential suffering may hinder adequate pain management. Furthermore, managing expectations throughout this process while maintaining hope may require a delicate balancing act, which should be addressed by specialized palliative care teams.
